# Applications of Thermochemical Modeling in Molten Salt Reactors

**DOI:** 10.3390/ma17020495

**Published:** 2024-01-20

**Authors:** Theodore M. Besmann, Juliano Schorne-Pinto, Mina Aziziha, Amir M. Mofrad, Ronald E. Booth, Jacob A. Yingling, Jorge Paz Soldan Palma, Clara M. Dixon, Jack A. Wilson, Donny Hartanto

**Affiliations:** 1 Nuclear Engineering Program, Department of Mechanical Engineering, University of South Carolina, Columbia, SC 29208, USA; julianos@mailbox.sc.edu (J.S.-P.); maziziha@mailbox.sc.edu (M.A.); amirmehdi.mofrad@sc.edu (A.M.M.); rebooth@sc.edu (R.E.B.); jakeying@gmail.com (J.A.Y.); claramd@email.sc.edu (C.M.D.); jackwilson@sc.edu (J.A.W.); 2 Oak Ridge National Laboratory, Oak Ridge, TN 37831, USA; hartantod@ornl.gov

**Keywords:** molten salts, redox, fission products, CALPHAD

## Abstract

The extensively evaluated and consistent thermodynamic database, the Molten Salt Thermal Properties Database—Thermochemical (MSTDB-TC), was used along with additional thermodynamic values from other sources as examples of ways to examine molten salt reactor (MSR) fuel behavior. Relative stability with respect to halide potential and temperature for likely fuel and fission product components were mapped in Ellingham diagrams for the chloride and fluoride systems. The Ellingham diagrams provide a rich, visual means for identifying halide-forming components in proposed fuel/solvent salt systems. Thermochemical models and values from MSTDB-TC and ancillary sources were used in global equilibrium calculations to provide compositions for a close analysis of the behavior of a possible Molten Chloride Salt Fast Reactor and a Molten Salt Reactor Experiment-type system at high burnup (100 GWd/t). The results illustrated the oxidative nature of burnup in MSRs and provided information about redox behavior and possible control.

## 1. Introduction

Molten salt reactors (MSRs) are Generation IV alternative systems whose attractive attributes have resulted in considerable international interest, with systems being developed in several countries. Key information for the design, operation, and regulation of MSRs relates to salt chemical behavior, including understanding and predicting phase formation, melting temperatures, corrosion mechanisms, and vaporization behavior. A salt chemistry roadmap [1] for the U.S. Department of Energy MSR program lists the various conceptual coolant and fuel salt melt compositions, which include both chloride and fluoride systems.

In support of MSR development, there has been an ongoing effort to generate and make widely available a thermochemical database of salt systems, allowing the equilibrium state of complex, multicomponent salts to be computed. This Molten Salt Thermal Properties Database—Thermochemical (MSTDB-TC) [2,3], together with a companion thermophysical database (MSTDB-TP) represent an important resource. The MSTDB-TC is intended to contain Gibbs energy relations for the solid, melt, and vapor species, including complex solid and liquid solutions, that allow properties to be computed using both commercial and open-source codes. Other than the possible short-lived effect of radiolysis, it is reasonable to assume that salt melts can be described by their thermodynamic equilibrium state, as their elevated temperatures and high species mobility in the liquid encourage approaching equilibrium.

The behavior of fission products and transuranic elements produced in prospective MSRs have received only modest attention. This is not surprising as gaining a sufficient understanding of the solvent salt systems together with fuel/fertile actinide elements (i.e., uranium, plutonium, and thorium) is itself a significant challenge. Individual fission products and transuranic elements will always be present at low concentrations, and in some respects, neglecting their behavior is not problematic. However, even at low levels, the radionuclides are seen as health and environmental hazards in the event of release in an accident. Benes et al. [4] illustrated the issue in their efforts to measure and compute vapor pressures generated from cesium- and iodine-containing components dissolved in a LiF–ThF_4_ melt.

Redox control in MSRs is seen as critical for managing corrosion potential and assuring that metallic actinide phases will not precipitate [5,6]. While the consequences of the failure to control redox potential for corrosion, as well as means for redox control have been investigated, there is still a need to understand its evolution during burnup.

The applications here discussed represent examples of the types of system analyses that the availability of thermochemical information makes possible. In addition to the described stand-alone equilibrium state calculations, they can also be utilized in MSR modeling and simulation codes that deal with mass accountancy, species transport, time-dependent corrosion, and other processes. These can include an equilibrium solver, such as Thermochimica [7] utilizing MSTDB-TC, which can be called to provide equilibrium state phase compositions, vapor pressures, and chemical potentials to the broader MSR code.

## 2. Assessing Relative Chemical States

While the fission process can create more than 60 different elements, their yield is characterized by the well-known bimodal distribution in atomic mass. That distribution shifts somewhat with the nature of the fissioning nucleus (^235^U, ^233^U, ^239^Pu) and with the neutron energy spectrum, yet the higher yield elements are largely the same. The subset of fission products as well as actinide elements and their likely oxidation states in fuel-salt systems are listed in Table 1. Also of interest are the elemental components of MSR structural alloys, as at least some of these will inevitably also appear as solutes in the melt, including iron, nickel, and chrome.

### 2.1. Ellingham Diagrams

**Figure 1:**
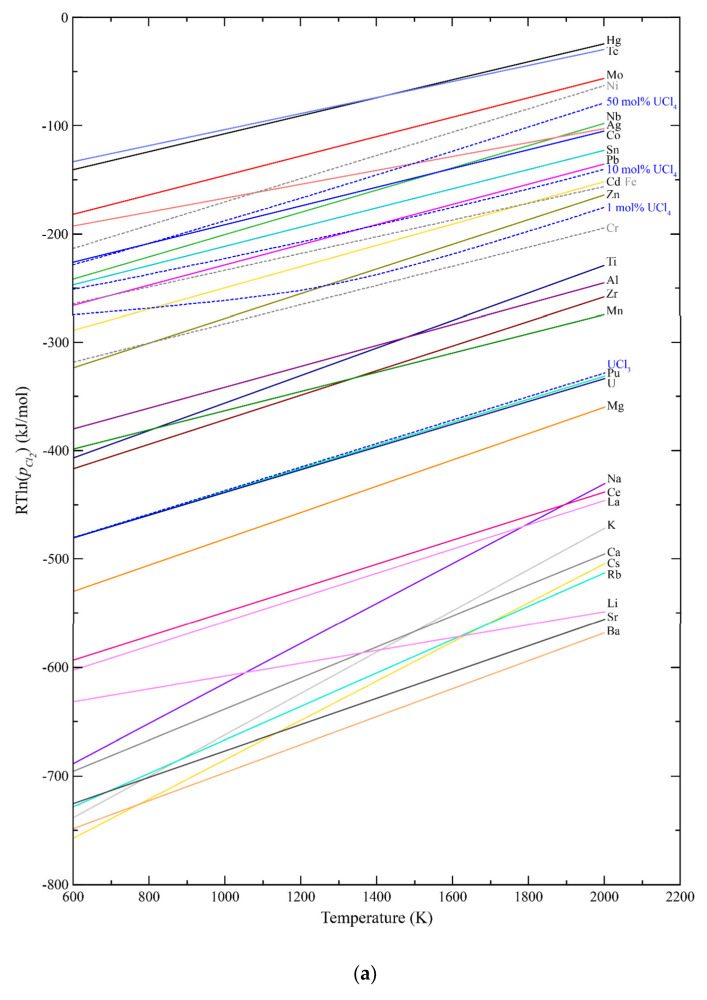

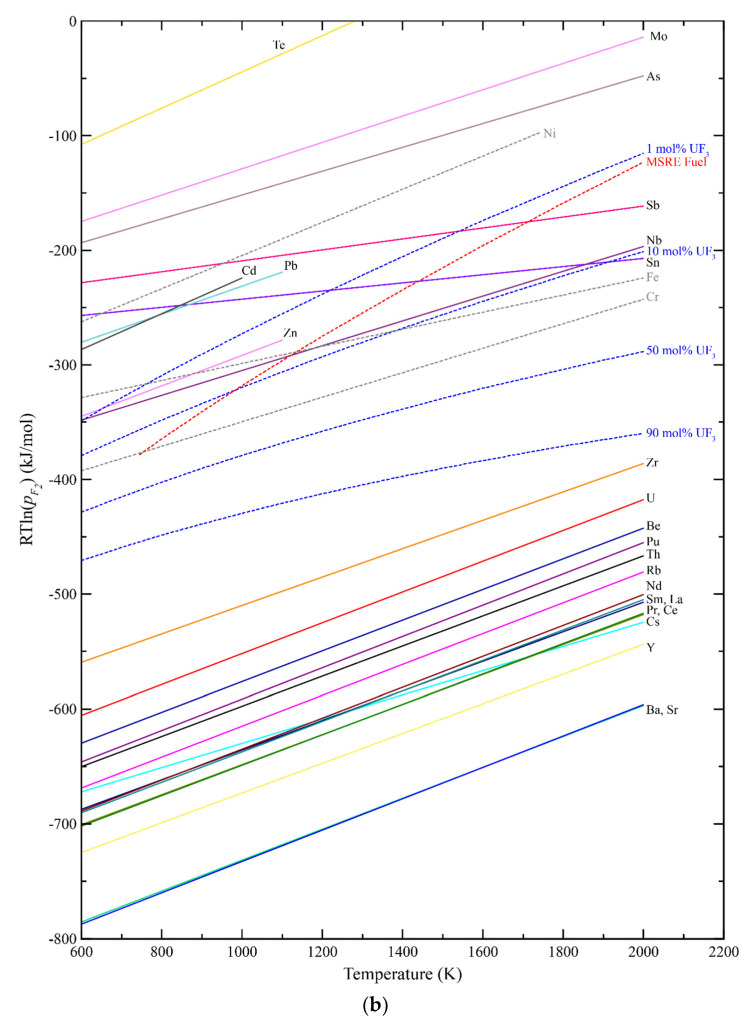
(**a**) Ellingham diagram for chloride salt fuel and fission products also indicating values for 1 mol%, 10 mol%, and 50 mol% UCl_4_ in UCl_3_ fuel salt. (**b**) Ellingham diagram for fluoride salt fuel and fission products also indicating values for 1 mol%, 10 mol%, 50 mol%, and 90 mol% UF_3_ content in UF_4_ fuel salt. Also indicated is the computed curve for MSRE fresh fuel salt.

One of the most simple and useful tools available for understanding relative stability among compounds having a common anion has been the Ellingham diagram [8]. It provides within a single plot the behavior of anion potential versus absolute temperature, and in the case of salts, the temperature dependence of the equilibrium relation between the cation and its lowest halide stoichiometry (or between lower and higher adjacent halides). The equilibria are represented by:

yM_(s,l)_ + X_2(g)_ = M_y_X_2(s,l)_

where M is a cation, X is a halogen, and y depends on the cation valence and thus the stoichiometry of the halide, with the components in their standard state. Ellingham diagrams for the chlorides and fluorides of cations of interest were generated utilizing Gibbs functions for the individual elements and the liquid salts from MSTDB-TC [2,3], Barin [9], and the SGTE salt database [10], and are seen in Figure 1. The delineated equilibrium relations of the diagrams are computed assuming the salt is a liquid, even though many of the individual salts are solids at the lower temperatures. The assumption that they are solely liquid across the temperature range, however, best represents their state as low concentration solutes in the solvent salt which is liquid at relatively low temperatures. Also, for convenience, rather than plotting the actual halide chemical potential values represented by the Gibbs formation energy for the individual halides, values of RTln (p_i_) are plotted for the equilibria between each metal and its halide, where R is the ideal gas law constant, T is absolute temperature, and p_i_ is the equilibrium vapor pressure of the halogen i.

The diagram of Figure 1a illustrates that even assuming only 1 mol% UCl_4_ in UCl_3_ fuel salt, a large majority of the metallic fission products as well as plutonium will be present as chloride salts. The metal–metal chloride equilibria for the likely structural alloy constituents chromium, iron, and nickel are also indicated.

The thermochemical relations among the fluoride systems are seen in the Ellingham diagram of Figure 1b, and in the case of fluoride fuel salts, uranium is expected to be predominantly UF_4_ while containing ~1 mol% UF_3_. Under these conditions, the actinides and majority of metallic fission products are indicated to form fluorides.

### 2.2. Thermochemical Modeling of Salt Systems

While Ellingham diagrams are extremely useful for quickly assessing the likely state of cationic elements, they provide a relatively simplistic picture of the thermochemical state of a salt-fueled MSR, both at startup and after significant burnup. A more realistic representation requires the use of accurate multicomponent thermochemical models of the salt and other materials. To perform such calculations, MSTDB-TC provides thermodynamic functions to a Gibbs energy minimizer code for computing equilibrium states. The database can be used directly with the FactSage^TM^ Ver. 8.2 [11] commercial software package and with the open-source codes Thermochimica [7] and PyCalphad [12].

It is challenging to represent salt melts due to their tendency to exhibit short-range ordering (SRO), preventing the use of more typical solution models [13]. The salt models utilized in MSTDB-TC therefore adopted the widely accepted modified quasi-chemical model in the quadruplet approximation (MQMQA) [13,14], which through the incorporation of coordination number information, can reasonably capture SRO behavior. The use of MQMQA for the MSR salt systems of MSTDB-TC is extensively discussed by Besmann et al. [3], Ard et al. [2] and Yingling et al. [15].

In addition to modeling the molten salt, it is necessary for MSTDB-TC to contain Gibbs energy functions for relevant crystalline salt phases and vapor species to fully define systems, and thus those are included. Stoichiometric phases and vapor species naturally have Gibbs energy functions that vary solely with temperature. Solid solution/variable composition phases are represented in the database by two-sublattice models exhibiting dependence on both composition and temperature [13].

The current MSTDB-TC ver. 3.0 contains evaluated thermochemical properties for 262 fluoride, chloride, and iodide pseudo-binary and -ternary, as well as higher order systems (details regarding database content and how to access the publicly available database can be found at https://mstdb.ornl.gov). This includes uranium, plutonium, and many of the important fission product elements as well as halide systems with chromium, iron, and nickel to help represent corrosion. Many of the system models are from novel evaluations that are documented in the materials that accompany the database.

## 3. Examples of MSR System Analyses

A chloride and a fluoride MSR concept were each chosen to provide examples of information that can be obtained from thermochemical analyses: The Molten Chloride Salt Fast Reactor (MCSFR) [16] and the historic Molten Salt Reactor Experiment (MSRE) [17].

### 3.1. MCSFR at High Burnup

The MCSFR is a two-fluid reactor designed to operate at 6000 MWth using NaCl carrier salt. The flowing fuel salt is (U/Pu)Cl_3_-NaCl, whereas the blanket salt is UCl_3_-NaCl. To obtain burnup fuel compositions, depletion calculations were carried out after the approach of Rykhlevskii et al. [18], using the SCALE/TRITON code [19] with a 2D cell model and the 238-group ENDF/B-VII.0 nuclear data library. To represent the two fluids, the 2D cell model has a cylindrical fuel salt channel with a thin outer layer of fertile salt enclosed within a square block of structural material (Hastelloy-N). Depletion calculations were performed assuming the fresh fuel composition of Table 2 for a high burnup of 100 GWd/t. A list of the higher concentration elements computed to be generated and considered in this analysis is seen in Table 3.

Equilibrium calculations for the system at a burnup of 100 GWd/t were performed using the FactSage^TM^ Ver. 8.2 [11] software, although several of the elements in Table 3 are not included in salt models in MSTDB-TC ver. 3.0. Since the missing elements have characteristics very similar to specific included elements, it was reasonably assumed that they can be represented by those elements. Thus, the amounts of each of the elements not included in the database were added to those of the appropriately analogous elements: Neptunium and americium amounts were added to that for uranium; promethium added to plutonium; gadolinium and yttrium added to neodymium; and praseodymium, samarium, and europium added to cerium. Gibbs energy functions for the tin and selenium phases were obtained from a FactSage^TM^ [11] internal database that, in turn, cites source references [9,20].

The results of equilibrium calculations for the 100 GWd/t composition at 1000K indicate that all the elements will be present as solute salts in the melt except for tin and selenium, which formed the SnSe phase along with elemental tin. The chlorine vapor pressure is also computed and was used in $RTln(p_{Cl_{2}})$ to obtain a value of −262 kJ/mol at 1000K. As expected, actinide fissioning is oxidative with the value having evolved from −316 kJ/mol computed for the fresh fuel. Also computed was the temperature at which the first salt precipitate NaU_2_Cl_7_ phase might form, which is 814K for the MCSFR at high burnup.

### 3.2. MSRE at High Burnup

The assumed MSRE fresh fuel composition was reported as that for the MSRE zero-power first ^235^U critical experiment [21] (Table 4), with the likely unintended amounts of oxygen, chromium, iron, and nickel from atmospheric contamination and corrosion of container material, and the trace hafnium content from uranium processing. The elemental quantities were used in equilibrium state calculations at 1000K using FactSage^TM^ Ver. 8.2 [11], MSTDB-TC plus thermochemical library values from Barin [9] and Chase et al. [22], yielding the melt species concentrations of Table 4. The additional phases ZrO_2_ and HfO_2_ (all hafnium is present as the oxide) were generated due to the oxygen content of the salt with the iron and nickel remaining metallic.

Depletion calculations to 100 GWd/t were performed for the MSRE configuration using the fresh fuel composition of Table 4 with the SCALE/TRITON code [19]. The burnup is substantially higher than the MSRE experienced, but is again being used to better understand how fuel evolves during the reactor operation. The resulting elemental amounts for the more abundant fission products plus base salt are seen in Table 5, with elements missing from MSTDB-TC aggregated with similarly behaving elements as described above for the MCSFR case.

Equilibrium calculations were performed using FactSage^TM^ Ver. 8.2 [11] with MSTDB-TC ver. 3.0, and the composition of Table 5. Non-salt crystalline phase Gibbs energy functions from an internal software database that were obtained from a variety of sources [9,22,23,24] were necessarily considered in the calculations.

The results of the equilibrium calculations for the 100 GWd/t composition at 1000K indicate that all the elements will be present as solute salts in the melt, except for small amounts of water vapor and the minor phases U_3_O_8_, ZrO_2_, and SrUO_4_, where essentially all the strontium is found in the latter phase. The computed fluorine vapor pressure at 1000K was used to obtain an $RTln(p_{F_{2}})$ value of −425 kJ/mol, with actinide fissioning again seen as oxidative as the value evolved from −647 kJ/mol computed for the fresh fuel. Also computed was the temperature at which a first salt precipitate might form, which is 740K for the Li_2_ZrF_6_ phase. The iron contaminant phase FeF_3_, however, precipitates just above that temperature at 745K.

## 4. Redox Potential

The operation of molten salt-fueled reactors requires the ability to determine and control the redox potential of the salt. Overly oxidizing conditions will cause rapid corrosion of structural materials (e.g., dissolution of alloying elements, most notably chromium which has the most negative halide formation energy). This is evident in the Ellingham diagrams of Figure 1, where values of RTln(p_i_) that are more positive than those for structural alloy components will drive their dissolution in the salt. Conversely, salts that are too reducing threaten to precipitate metallic phases (in addition to the noble metals), such as uranium. Redox potential can also influence other properties, including species vapor pressures, and these too can be computed from the overall thermochemical state of the salt.

As noted, calculations for both the MSCFR and MSRE indicate burnup will cause the salt to become more oxidizing. Indeed, the RTln(p_i_) values for chlorine or fluorine in the reactor systems at 1000K and a burnup of 100 GWd/t are more positive than the values for the Cr–CrX_2_ equilibria, and thus there is a thermodynamic driving force to cause chrome dissolution from the structural alloy.

The need for controlling redox was well recognized during the development and operation of the MSRE. Thoma [25] noted in his assessment of the chemistry of the MSRE operations that both a real-time measure of redox potential plus a means for controlling it are necessary, with a specific goal of keeping the UF_3_ content ≤0.05 mol% to prevent removing chromium from the Hastelloy N structural material. Extended MSRE operations required the addition of metallic beryllium to reduce the fluorine potential that was increasing during burnup [25].

The chloride fuel-related Ellingham diagram of Figure 1a contains the line for not only for the U–UCl_3_ equilibria but also with added UCl_4_ (1 mol%, 10 mol% and 50 mol%) providing guidance as to controlling the redox potential by controlling the ratio of chlorides. Similarly, Figure 1b provides values for UF_4_ with various concentrations of UF_3_ (1 mol%, 10 mol%, 50 mol%, and 90 mol%) as well as the computed curve for the MSRE fresh fuel composition of Table 4.

## 5. Conclusions

The now available thermodynamic database MSTDB-TC ver. 3.0 allows for detailed equilibrium calculations for molten salt fuel systems and can be used together with databases containing Gibbs energy functions for other materials, such as structural alloys, to begin to realistically simulate MSR chemical behavior. A useful set of Ellingham diagrams was generated that allows for a quick, visual determination of the relative stability of metallic materials and their salts and their dependence on fuel composition.

A detailed set of equilibrium calculations were performed using salt compositions at high burnup (100 GWd/t) utilizing the MCSFR concept and the historical MSRE system. These provided an illustration of how salt chemistry evolves during burnup, offering a quantitative understanding of chemical behavior.

While the results reported here provide a means for obtaining a better understanding of complex MSR salt systems, care must be taken in their application. Ellingham diagrams offer useful insights, yet it should be recognized that these provide a simplistic view of overall chemical behavior. For example, the lines for each metal–metal halide are generated without consideration of the influence of the solvent salt on the equilibria. In addition, although the values for RTln(p_i_) may be in a range where, from a thermodynamic point of view, structural alloy components would not be expected to form halides and dissolve in the salt melt, entropic driving forces will always result in some dissolution.

Global thermochemical calculations obtained using MSTDB-TC can provide accurate results, yet these are time-independent determinations that do not consider kinetic factors or material transport. Thus, for example, in simulating corrosion mechanisms and determining corrosion rates, much more complex phenomena need to be represented, as well described by Bhave et al. [26] and Pillai et al. [27], among others.

Ultimately, thermochemical analyses can provide the basis for understanding complex, multicomponent reacting systems that approach equilibrium, and provide guidance and input for simulating transport and other time-dependent processes. Future thermochemical efforts are focusing on using equilibrium calculations to compute species vapor pressures over complex salt compositions and realistic solubilities of corrosion products in salts in equilibrium with structural alloys. These are in addition to the continuing effort to refine and expand MSTDB-TC.

## Figures and Tables

**Table 1:** Most abundant fission products and actinide elements and their likely oxidation states in a halide salt-fueled MSR.

Element	Salt Melt Constituents
Actinide	Th^4+^ U^3+,4^ Pu^3+^ Np^3+^ Am^3+^ Cm^3+^
Alkali	Li^+^ Na^+^ K^+^ Rb^+^ Cs^+^
Alkaline earth	Be^2+^ Mg^2+^ Ba^2+^ Sr^2+^
Transition metal	Zr^4+^ Y^3+^ Sn^2+^ Cd^2+^ Hf^4+^ In^3+^ As^3+^ Ge^4+^ Ta^4+^ Mn^2+^ Mo^+5^ Nb^3+^ Co^2+^ Te^4+ 2-^ Sb^3+ 3-^ Ga^2+^ Zn^2+^ W^5+^ Ag^-^ Hg^+1^ Pb^2+^ Bi^3+^
Lanthanoid	La^3+^ Ce^3+^ Pr^3+^ Nd^3+^ Pm^3+^ Sm^3+^ Eu^3+^ Gd^3+^ Tb^3+^ Dy^3+^ Ho^3+^ Er^3+^ Tm^3+^ Yb^3+^ Lu^3+^
Halide	Cl^-^ Br^-^ I^-^
Noble metals	Tc Pd Rh Ru Mo Ag
Inert gases	He Ne Kr Xe

**Table 2:** Fresh fuel composition assumed for the MCSFR.

Fuel Component	Mol%
NaCl	60.00%
UCl_3_	35.46%
PuCl_3_	4.54%

**Table 3:** Higher concentration elements in the fuel salt at 100 GWd/t for the MCSFR.

Element	At.%	Element	At.%	Element	At.%	Element	At.%	Element	At.%
Cl	63.10	Zr	0.350	La	0.100	Kr	0.0326	Pm	0.0121
Na	21.03	Cs	0.349	Tc	0.0961	I	0.0290	He	0.0102
U	10.58	Nd	0.291	Pr	0.0896	Rb	0.0283	Sn	0.00978
Pu	1.655	Pd	0.273	Sm	0.0767	Ar	0.0259	Eu	0.00927
Xe	0.424	Ce	0.192	Sr	0.0634	Ag	0.0208	Gd	0.00720
Mo	0.399	Ba	0.136	Te	0.0553	Cd	0.0152	Se	0.00682
Ru	0.351	Rh	0.107	Y	0.0343	Mg	0.0144	Np	0.00674
								Am	0.00357

**Table 4:** Fresh MSRE fuel elemental composition [21] and computed melt species at 1000 K.

Fuel Analysis (Element)	At.%	Equilibrated Melt Species	At.%
F	59.41	LiF	64.91
Li	26.30	BeF_2_	29.28
Be	11.86	ZrF_4_	4.99
Zr	2.049	UF_4_	0.783
U	0.319	UF_3_	0.0055
O	0.052	CrF_2,3_	0.012
Cr	0.0049	FeF_2_	0.008
Fe	0.0049		
Ni	0.00086		
Hf	0.000052		

**Table 5:** Higher concentration elements in MSRE fuel salt at 100 GWd/t (at.%).

Element	At.%	Element	At.%	Element	At.%	Element	At.%	Element	At.%
F	59.41%	O	0.052%	Ce	0.0042%	La	0.0021%	Ni	0.0009%
Li	26.30%	Nd	0.0064%	He	0.0036%	Pr	0.0019%	Rb	0.0008%
Be	11.86%	Fe	0.0049%	Cs	0.0024%	Y	0.0015%	H	0.0007%
Zr	2.06%	Cr	0.0049%	Sr	0.0024%	Sm	0.0010%	Np	0.0002%
U	0.28%	Pu	0.0044%	Ba	0.0023%				

## Data Availability

Data are available within the article or within the MSTDB-TC Ver. 3.0 database which can be accessed as described in the article.
